# Effect of dietary lutein on the egg production, fertility, and oxidative injury indexes of aged hens

**DOI:** 10.5713/ab.22.0473

**Published:** 2023-05-02

**Authors:** N. Liu, X. Ji, Z. Song, X. Deng, J. Wang

**Affiliations:** 1 Department of Animal Science, Henan University of Science and Technology, Luoyang, Henan 471000, China; 2 National Engineering Research Center of Biological Feed, Beijing 100081, China

**Keywords:** Aged Hen, Egg Production, Fertility, Lutein, Oxidative Injury Index

## Abstract

**Objective:**

The present study aimed to investigate the effect of dietary lutein on egg production, follicles, reproductive hormones, fertility, hatchability, and oxidative injury indexes of hens.

**Methods:**

Treatments consisted of a control diet (CON) and three lutein-supplementing diets at 25 (L1), 50 (L2), or 75 (L3) mg/kg of diet. Egg production was measured using 576 Arbor Acres breeder hens at 61 to 65 wk and follicles grades, reproductive hormones, fertility, hatchability, tissue lutein contents, and oxidative injury indexes were determined at 65 wk.

**Results:**

The results showed that at 65 wk, lutein-supplementing diets increased (p<0.05) egg production, follicular grades, fertility, hatchability, estradiol (E2), luteinizing hormone, progesterone (PROG), lutein content in the serum and yolk, compared to CON. L2 and L3 showed more pronounced (p<0.05) effects on egg production, PROG, and yolk lutein content than L1. With the increase of lutein doses from 25 to 75 mg/kg, there were linear increases (p<0.05) in egg production, lutein content, and PROG, and a quadratic trend (p<0.05) in E2. For the oxidative injury products, lutein-supplementing diets decreased (p<0.05) malondialdehyde (MDA) and protein carbonyl (PCO) in the serum, MDA and 8-hydroxy 2 deoxyguanosine (8-OHdG) in the yolk. There were linear decreases (p<0.05) in 8-OHdG in the serum, MDA, PCO, and 8-OHdG in the yolk, a quadratic trend (p< 0.05) on serum 8-OHdG.

**Conclusion:**

It is concluded that lutein supplementation can improve egg production and fertility by beneficially regulating reproductive hormones and oxidative status in aged hens.

## INTRODUCTION

Lutein is a xanthophyll carotenoid with an orange color, rich in some plants [1]. Commercially, marigold flowers are a primary source of lutein, and the marigold lutein is often used as a coloring agent in food and feed [2]. Besides that, lutein as an eye pigment is essential for humans and animals that are unable to synthesize [3]. More importantly, lutein is a critical antioxidant for plants and animals [4]. Lutein in the body tissues was shown to have beneficial effects on the visual, nervous, cardiovascular, urinary, and immune systems [5,6]. Several latest studies reported that lutein improved oocytes, embryo quality, fertilization rate, and 2-cell blastocyst in the polycystic ovary syndrome model of mice [7,8]. Lutein increased cumulus expansion, oocyte viability, and embryo developmental potential to the morula stage by protecting lipid components in oocytes and embryos against oxidative injury in swine [9]. Additionally, during egg incubation, the antibacterial capacity of egg lysozyme was highly dependent on yolk lutein content [10]. However, studies showed that dietary lutein added at 10 to 40 mg/kg of diet had no significant effect on the egg production of hens [11,12].

The findings related to reproduction raise the possibility that lutein can be vertically transmitted from mother to offspring. The vertical transmission may be necessary in improving the fertility and hatchability of breeders, but data are scarce. Additionally, aged broiler breeder hens have poorer egg production and fertility than other species, which is a technical constraint in practice. Based on the knowledge between lutein and reproduction, the present study aimed to test the hypothesis that supplementing lutein to the diet can improve follicular development, egg production, fertility, hatchability, and reproductive hormones, and decrease oxidative injury in aged broiler hens.

## MATERIALS AND METHODS

### Animal ethics approval

Research on animals was approved by institutional committee on animal use in Henan University of Science and Technology (No. 2021002).

### Lutein and diets

Lutein was extracted from marigold flowers. A basal diet (control, CON) contained white corn and soybean meal as main ingredients. The lutein was added at 25 (L1), 50 (L2), and 75 mg/kg (L3) of diet using step by step enlargement method. The determined lutein contents in the CON, L1, L2, and L3 were 0.26, 24.37, 49.02, and 74.28 mg/kg, respectively. The basal diet was formulated referring to the nutrient requirements of Arbor Acres parent stock Handbook (Aviagen, 2018). The ingredients, chemical levels, and lutein contents in diets are listed in Table 1.

### Animals and samples

A total of 576 Arbor Acres parent stock hens at 60 wk of age without statistical differences in egg production and body weights were randomly assigned to four dietary groups. Each treatment contained six replicates of 24 hens in two-tiers cages. All replicates were uniformly distributed in a closed chicken house to minimize the environmental effect [13]. All hens were supplied with feed at 135 g/hen/d and *ad libitum* water in the house with auto-ventilation, lighting system 16L/8D (light/dark) and temperature 20°C according to the management manual of Arbor Acres parent stock hens. After a week of adjustment, the feeding trial lasted from 61 to 65 wk of age. Artificial insemination was administered once every five days. Eggs and mortality per replicate were recorded daily and egg laying rate was calculated weekly [14]. Hen’s health was monitored twice daily.

At the last trial day, eight hens per replicate were randomly selected and wing vein blood was collected to analyze hormones, oxidative products, and lutein content [15]. The eight hens were euthanized by carbon dioxide and dissected, and follicular diameters were measured at two grades, large follicles (Φ≥15 mm) and middle follicles (3≤Φ<15 mm). At the last week of feeding trial, 300 eggs per replicate were incubated to determine fertility and hatchability. At day 7 of incubation, candling inspection was carried out to calculate the fertility (%, fertilized/total hatched eggs). At day 21, chicks were sorted out and hatching rate (%, chicks/total fertilized eggs) were calculated. A total eight yolks per replicate were used to determine lutein and oxidative product content.

### Chemical and biochemical analysis

Lutein content in the marigold product and diets were measured by high performance liquid chromatography equipment (Agilent 1260; Agilent Technologies, Santa Clara, CA, USA) according to the method of China National Standard GB 26405-2011. The detection conditions were set as detector wavelength 446 nm, silica gel column 4.6 mm×250 mm (size, 3 μm), the ratio of hexane to ethyl acetate 70/30 (v/v), flow rate 1.5 mL/min, and injection volume 10 μL at room temperature. The spike recovery of serially diluted lutein was 96.2% to 105.7%.

The concentrations of hormones and oxidative products were measured using commercial kits from Nanjing Jiancheng Biological Institute (Nanjing, China) for estradiol (E2; H102-1), follicle-stimulating hormone (FSH; H101-1-2), luteinizing hormone (LH; H206-1-2), prolactin (PRL; H095-1-2), progesterone (PROG; H089), malondialdehyde (MDA; No. A003), protein carbonyl (PCO; No. A087), and 8-hydroxy 2 deoxyguanosine (8-OHdG, No. H165). The unit of MDA, PCO, and 8-OHdG were expressed as per gram protein in the yolk samples to minimize the errors during sample preparation [16]. During the chemical and biochemical analysis, all samples were determined in triplicate.

### Statistical analysis

Data are represented as mean and SEM using SPSS software (version 23; IBM SPSS, Armonk, NY, USA). Differences between mean values of normally distributed data were assessed with one-way analysis of variance (Tukey test) at p<0.05 level of significance. The statistical unit was the mean of 24 hens for egg production, of eight hens for follicles, hormones, and oxidative injury indexes in the serum, of 300 eggs for fertility and hatchability, and of eight yolks for lutein contents and oxidative injury indexes. The variable responses to the lutein doses of 25, 50, and 75 mg/kg were analyzed using contrasts of linear and quadratic polynomials.

## RESULTS

### Effect of lutein on egg production and follicular grades

**Figure 1:**
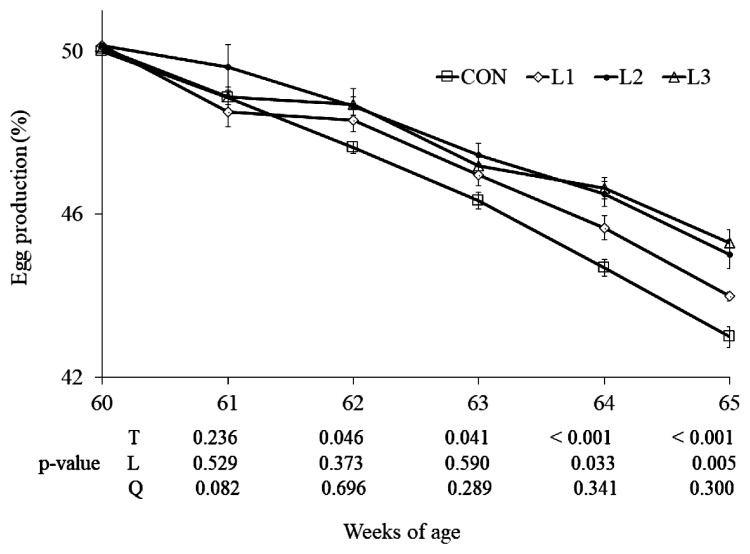
Effect of dietary lutein on the egg production in Arbor Acres breeder hens (mean and standard error). Lutein is added at 0 (CON, control), 25 (L1), 50 (L2), and 75 (L3) mg/kg of diet, respectively, from 61 to 65 wk of age. T, treatment effect; L and Q, linear and quadratic effect of marigold lutein doses from 25 to 75 mg/kg.

**Figure 2:**
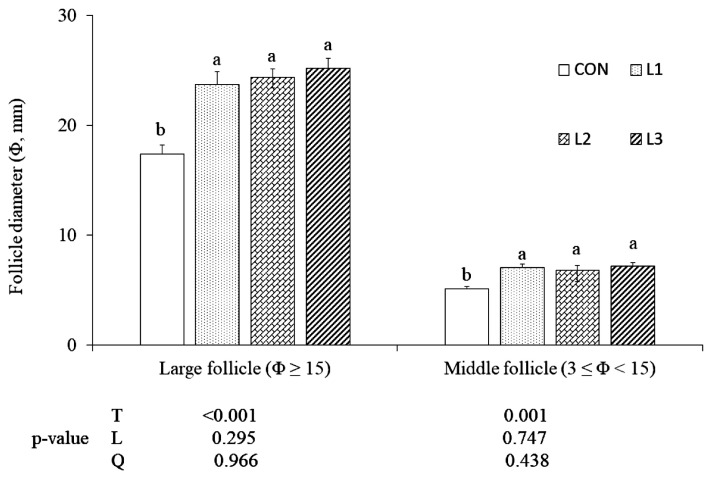
Effect of lutein on the follicle diameter (Φ) of Arbor Acres breeder hens at the 5th week of feeding trial (65 wk of age, mean and standard error). Lutein is added at 0 (CON, control), 25 (L1), 50 (L2), and 75 (L3) mg/kg of diet, respectively, from 61 to 65 wk of age. T, treatment effect; L and Q, linear and quadratic effect of marigold lutein doses from 25 to 75 mg/kg of diet. ^a,b^ Means among treatments without the same superscripts are significantly different (p<0.05).

From 62 wk of age, there were significant treatment effects (p<0.05) on the egg-laying rate (Figure 1). As the lutein doses increased from 25 to 75 mg/kg, there were linear increases (p<0.05) in egg production at 64 to 65 wk. Additionally, just two hens died in the experimental period, because of this, the mortality rate was so low and data were not shown. At 65 wk of age, the average diameters of large and middle follicles in the three lutein treatments were greater (p<0.05) than CON, but there were no differences among the three lutein treatments (Figure 2).

### Effect of lutein on fertility, hatchability, and reproductive hormones

The lutein-containing diets increased (p<0.05) fertility and hatchability by 4.1% and 5.9%, respectively, compared to CON (Table 2). The lutein treatments also had the higher levels (p<0.05) of E2, LH, and PROG than CON. L2 and L3 increased (p<0.05) FSH compared to CON. The three doses of lutein showed increments (p<0.05) in PROG. The average increases of E2, FSH, LH, and PROG in lutein treatments were 64.4%, 85.4%, 64.5%, and 38.6%, respectively. Additionally, as the lutein doses increased, there was a linear response (p<0.05) for PROG and a quadratic response (p< 0.05) for E2.

### Tissue lutein contents and oxidative injury indexes

The dietary lutein increased (p<0.05) and its deposition in the serum and yolk compared to CON (Table 3); L2 and L3 had a more pronounced effect (p<0.05) than L1 on the yolk lutein. With the increased lutein doses in diets, linear increases (p<0.05) were found in serum lutein and yolk lutein.

In the serum, the three lutein diets decreased (p<0.05) MDA and PCO compared to CON; only L2 and L3 showed a decreasing effect (p<0.05) on 8-OHdG; and with the lutein doses, there were linear and quadratic decreases (p<0.05) in 8-OHdG. In the yolk, the three lutein diets decreased (p<0.05) MDA and 8-OHdG; but only L3 decreased (p<0.05) PCO; the decrease effect of L3 on 8-OHdG was more pronounced (p<0.05) than L1 and L2; linear effects (p<0.05) were found on MDA, PCO, and 8-OHdG.

## DISCUSSION

Lutein is synthesized by plants and is an important antioxidant in nature. Currently, the egg industry uses dietary lutein to vary yolk color according to consumer preference. Besides that, ingested lutein circulates and is deposited in the body to exert an antioxidative effect in human and animal tissues. The antioxidative effect of lutein may be helpful to the egg production of hens, especially aged hens. Indeed, in the present study, the breeder hens fed with lutein at 25, 50, or 75 mg/kg had a higher laying rate than CON. However, this is inconsistent with the report that dietary lutein at 10, 20, 30, and 40 mg/kg had no significant effect on egg mass, feed intake, and egg weight of laying hens at 26 to 28 wk of age [11]. The aged hens have a relatively fragile physique and are more susceptible to dietary factors, which may partially explain the inconsistent effect of lutein on egg production between the present study and literature; however, this needs to be studied further. Additionally, egg mass, egg weight, and feed intake with less importance than egg number for aged breeder hens were not shown and discussed in the present study.

In avian species, egg production is mainly presupposed by follicular grades. Due to the substantial size of chicken follicles, the average diameter can be used to compare follicular grades. Follicular development involves a complex process which ultimately results in ovulation. At the same time, thousands of developing follicles undergo atresia. Studies showed that antioxidants strongly benefit follicular development and their size increase [12,14,17]. Carotenoids as one of the most important antioxidants in the nature were also shown to have effective antioxidation in the ovary. Literature reported that carotenoid lycopene decreased oxidative stress and improved the function of D-gal-induced and naturally aged ovaries [18]. Lutein increased the numbers of normal oocytes and oocyte quality in a polycystic ovary syndrome model [7]. Literature about dietary lutein’s effect on follicular development in poultry is scarce. In the present study, the average diameters of follicles were increased in lutein diets, compared to CON, indicating that lutein can improve follicle growth and maturation, and alleviate the ovarian degeneration of aged hens, but more studies are needed.

The antioxidative activity of lutein on follicular development and quality may also have a cascade effect on fertilization, embryonic development, and hatchability. Indeed, in the present study, dietary lutein improved the percentages of fertile eggs, embryos, and healthy chicks. Literature about this is minimal. Bandariyan et al [7] reported that lutein significantly increased fertilization and blastocysts and decreased arrested embryos. Since the antioxidant and immunostimulatory roles of carotenoids are critical during the immediate post-hatch period, maternal dietary intake of carotenoids exhibited important ramifications for the viability of chicks in the first week of age [19]. Data are scarce about the effect of carotenoids, including lutein, on the percentages of hatchability and healthy chicks. As known, there are two peak periods of death during the chicken embryo development, distributed at 2 to 4 and 19 to 21 d of the embryo. The first death peak is mainly related to the inner quality of eggs and the second is involved in external hatching conditions. How the maternal lutein incorporated in the egg affects the dynamic and peak death of chicken embryos deserves further study.

The reproductive behavior of hens is strictly regulated by reproductive hormones, such as E2, FSH, LH, and PROG. Certain levels of these hormones are necessary to maintain egg production. In theory, these hormones are naturally secreted and fluctuate with physiological phases in a reproductive cycle. Non-hormonal external factors, such as dietary manipulation and environmental changes, can only have a slight impact on these hormones. However, the improvement in egg production from dietary manipulation can make a sizable profit for large-scale intensive output, especially for commercially important broiler chicken or turkey breeders who genetically have poor reproduction [12,14]. In the present study, the relatively high levels of E2, FSH, LH, and PROG in lutein diets may partially explain the increased effect on egg production. Literature about the direct relationship between dietary lutein and reproductive hormones is scarce. Indirectly, lutein ameliorated reproductive damage by alleviating oxidative stress, inflammation, and apoptosis in male rats [8]. Serum antioxidants were associated with serum reproductive hormones and ovulation among healthy women [20]. Anyway, whether the lutein supplementation and reproductive release reflect a cause and effect relationship remains to be further determined.

Oral lutein enters the bloodstream, travels throughout the body, incorporates into some tissues, and influences the body’s health. Getting back to the fundamentals, all of the health benefits of lutein are the result of its antioxidative activity. Lutein presence at a particular concentration in the body prerequisites its antioxidation, and the effect, weak or strong, is putatively dependent on the concentration of lutein. Hens, by feeding lutein fortified *Chlorella*, significantly increased lutein contents in the serum, liver, growing oocytes, and eggs [21]. Similarly, lutein concentration varied from 0.04 to 0.68 μg/g in the eye, brain, heart, lung, intestine, pancreas, kidney, and breast of hens fed with a lutein-enriched diet [22]. In the present study, hens in lutein treatments had higher levels of lutein in the serum and yolk, and lower concentrations of oxidative products, including MDA, PCO, and 8-OHdG, indicating that the oxidative damages of the body were decreased in lutein diets.

Oxidative damage in the body is widespread, mainly manifested as damage to the structure and function of biological macromolecules, which leads to genetic mutations, cell carcinogenesis and individual aging. The MDA, PCO, and 8-OHdG are the oxidative products of lipids, proteins, and deoxyribonucleic acids, respectively. Lutein intervention decreased serum MDA and 8-OHdG in rats [23]. Carotenoid blends including lutein decreased MDA in broilers [24]. Lutein treatment blocked the high glucose-mediated elevation of intracellular reactive oxygen species, PCO, and MDA content in ARPE-19 cells [25]. Lutein protected against vancomycin-induced renal injury by decreasing MDA, PCO, and related inflammatory and apoptotic signal pathways [26]. The decreases in the oxidative terminal products of biological macromolecules by lutein in the present study and the literature demonstrated the protective effect of lutein in the body. Interestingly, lutein derived from marigold petals suppressed cancer cells by triggering reactive oxygen species generation and activating apoptotic genes [27]. The paradoxical responses of lutein to reactive oxygen species and cell survival in normal and abnormal cells needs further study.

## CONCLUSION

The dietary lutein improved the egg production, fertility, and hatchability of broiler breeder hens in the late reproductive stage. These benefits of lutein are related to lutein enrichment in the circulating system and critical tissues of the body, and the antioxidative property of lutein and its cascade on the reproductive hormones. It is concluded that lutein can be used as an antioxidant to improve the reproduction and hatchability of aged hens.

## Figures and Tables

**Table 1:** Composition and nutrient level of basal diet (as fed basis, %)

Items	Content
Ingredient	
White corn	64.6
Soybean meal	11.8
Peanut meal	5.0
Wheat bran	7.5
Salt	0.4
Methionine	0.2
Lysine	0.2
Dicalcium phosphate	1.3
Limestone	8.0
Premix^1)^	1.0
Nutrient	
Metabolizable energy^2)^ (MJ/kg)	11.03
Crude protein	14.02
Calcium	3.30
Non-phytate phosphorus	0.35
Methionine	0.40
Lysine	0.78
Sulfur-containing amino acids	0.62

1) Provided per kg of diet: vitamin A (retinyl acetate), 8,000 IU; cholecalciferol, 1,600 IU; vitamin E (DL-α-tocopheryl acetate), 5 IU; vitamin K, 0.5 mg; riboflavin, 2.5 mg; D-pantothenic acid, 2.2 mg; niacin, 20 mg; pyridoxine, 3.0 mg; biotin, 0.10 mg; folic acid, 0.25 mg; vitamin B_12_, 0.004 mg; choline, 500 mg; manganese, 60 mg; iodine, 0.35 mg; iron, 60 mg; copper, 8 mg; zinc, 80 mg; and selenium, 0.30 mg.

2) Calculated according to Chinese Feed Database (2019, 30th ed).

**Table 2:** Effect of dietary lutein on the fertility, hatchability, and reproductive hormones of aged hens at 65 wk of age

Item	Hatching performance		Reproductive hormone			
	
	Fertility (%)	Hatchability (%)	E2 (pg/mL)	FSH (IU/L)	LH (ng/mL)	PROG (pmol/L)
Treatment^1)^						
CON	79.5^b^	74.2^b^	133^b^	8.72^b^	58.7^b^	598^d^
L1	82.2^a^	78.0^a^	219^a^	14.4^ab^	91.4^a^	710^c^
L2	82.2^a^	78.5^a^	201^a^	16.1^a^	96.9^a^	832^b^
L3	83.8^a^	79.2^a^	236^a^	18.8^a^	101.3^a^	944^a^
SEM	0.689	0.735	9.416	1.573	4.156	15.67
p-value^2)^						
T	0.003	<0.001	<0.001	0.003	<0.001	<0.001
L	0.086	0.166	0.235	0.124	0.213	<0.001
Q	0.317	0.883	0.043	0.841	0.896	0.806

E2, estradiol; FSH, follicle-stimulating hormone; LH, luteinizing hormone; PROG, progesterone; SEM, standard error of the mean.

1) Lutein was added at 0 (CON), 25 (L1), 50 (L2), and 75 (L3) mg/kg of diet, respectively, from 61 to 65 wk of age.

2) T, treatment effect; L and Q, linear and quadratic effect of lutein doses from 25 to 75 mg/kg of diet.

a–d Means among treatments without the same superscripts are significantly different (p<0.05).

**Table 3:** Effect of dietary lutein on the tissue lutein and oxidative injury indexes of aged hens at 65 wk of age

Items	Lutein content		Oxidative injury index					

			Hen serum			Egg yolk		
		
	Serum (mg/L)	Yolk (μg/g)	MDA (mmol/L)	PCO (mmol/L)	8-OHdG (pg/mL)	MDA (nmol/g)	PCO (ng/g)	8-OHdG (pg/g)
Treatment^1)^								
CON	0.22^b^	6.6^c^	3.70^a^	0.56^a^	40.0^a^	0.40^a^	2.83^a^	28.8^a^
L1	0.31^a^	20.4^b^	2.45^b^	0.45^b^	35.6^a^	0.29^b^	2.57^a^	25.8^b^
L2	0.32^a^	29.4^a^	2.38^b^	0.43^b^	27.0^b^	0.27^b^	2.60^a^	25.2^b^
L3	0.34^a^	32.8^a^	2.35^b^	0.42^b^	27.3^b^	0.24^b^	2.25^b^	23.4^c^
SEM	0.013	0.975	0.097	0.013	1.332	0.012	0.068	0.387
p-value^2)^								
T	<0.001	<0.001	<0.001	<0.001	<0.001	<0.001	<0.001	<0.001
L	0.036	<0.001	0.395	0.056	<0.001	0.037	0.015	<0.001
Q	0.843	0.108	0.870	0.923	0.024	0.990	0.541	0.116

MDA, malondialdehyde; PCO, protein carbonyl; 8-OHdG, 8-hydroxydeoxyguanosine; SEM, standard error of the mean.

1) Lutein was added at 0 (CON), 25 (L1), 50 (L2), and 75 (L3) mg/kg of diet, respectively, from 61 to 65 wk of age.

2) T, treatment effect; L and Q, linear and quadratic effect of lutein doses from 25 to 75 mg/kg of diet.

a–c Means among treatments without the same superscripts are significantly different (p<0.05).
